# Hippo–YAP/TAZ Signaling in Astrocytes and Microglia: Role in Neuroinflammation, Neurodegeneration and Glial Tumors

**DOI:** 10.3390/ijms27083672

**Published:** 2026-04-20

**Authors:** Emilia Zgorzynska

**Affiliations:** Department of Cell-to-Cell Communication, Medical University of Lodz, Mazowiecka 6/8, 92-215 Lodz, Poland; emilia.zgorzynska@umed.lodz.pl

**Keywords:** Hippo signaling, YAP/TAZ, astrocytes, microglia, neuroinflammation, gliomagenesis, glioblastoma

## Abstract

Glial cells, particularly astrocytes and microglia, are central to maintaining CNS homeostasis and coordinating responses to injury through tightly regulated metabolic, inflammatory, and mechanosensitive processes. Emerging evidence identifies the Hippo signaling pathway and its downstream effectors YAP/TAZ as key regulators of glial functions, influencing proliferation, polarization, intercellular communication, and the balance between neuroprotection and neurotoxicity. This review discusses the Hippo signaling pathway and its transcriptional co-activators YAP/TAZ as context-dependent hubs integrating mechanical, metabolic, and immune cues in astrocytes and microglia. Particular attention is given to MST1/2- and YAP/TAZ-dependent signaling in microglia, which governs inflammatory states, redox balance, mitophagy, and mechanosensing. In astrocytes, Hippo–YAP signaling emerges as a bidirectional regulator of reactive gliosis and neuroprotection, capable of constraining excessive scar formation. However, when chronically suppressed, it impairs glutamate clearance, metabolic support, and resistance to neurodegeneration. Disruption of Hippo signaling in glial tumors is also considered, with YAP/TAZ–TEAD complexes driving glioblastoma stemness, infiltrative growth, immune evasion, and therapy resistance. Finally, therapeutic perspectives are outlined that emphasize context-selective modulation of Hippo signaling in the CNS. Overall, Hippo–YAP/TAZ signaling is presented as a highly context-dependent regulator at the interface of glial inflammation, neurodegeneration, and glioma biology and as a promising but demanding target for future CNS therapies.

## 1. Introduction

Glial cells constitute the major non-neuronal population in the central nervous system (CNS), encompassing astrocytes, microglia, and oligodendrocytes, collectively ensuring the maintenance of neural homeostasis and tissue integrity [1]. Among them, astrocytes and microglia play the most dynamic and adaptable roles, integrating metabolic, immune, and neurovascular processes that sustain neuronal function and brain health [2]. Astrocytes are the most abundant glial subtype in the CNS and are central to maintaining extracellular ion balance, glutamate clearance, and neurovascular coupling. They regulate synaptic transmission by uptaking neurotransmitters through excitatory amino acid transporters (EAAT1/EAAT2) and by buffering extracellular potassium ions. Moreover, astrocytes sustain neuronal energetics through the astrocyte–neuron lactate shuttle, modulate cerebral blood flow, and maintain blood–brain barrier (BBB) integrity [3]. In response to injury or disease, astrocytes display remarkable functional plasticity, a process referred to as reactive astrogliosis, characterized by cytoskeletal remodeling, transcriptional reprogramming, and altered secretory profiles [4,5]. While moderate activation serves neuroprotective and reparative functions, excessive or chronic activation contributes to neuroinflammation and neuronal dysfunction [6,7]. In turn, microglia, the brain’s tissue-resident parenchymal macrophages, serve as the brain’s primary innate immune effectors [8]. In the healthy brain, microglia continuously survey their environment, prune redundant synapses, clear debris, and release trophic factors that sustain neuronal health and network integrity [9]. Upon infection, trauma, or protein aggregation, microglia transition into reactive phenotypes characterized by metabolic reprogramming, increased cytokine production (IL-1β, TNF-α, IL-10), and alterations in mitochondrial dynamics [10].

The bidirectional communication between astrocytes and microglia is crucial for both maintaining brain health and responding to injury or disease [11,12]. Depending on the pathological context and the disease stage, microglia can promote anti-inflammatory and neuroprotective astrocyte phenotypes through the secretion of factors such as IGF-1, TGF-α, plexin-B2, and microRNA-124 [13,14,15,16]. In turn, activation of inflammatory pathways, including NF-κB, MAPK, PI3K, and Akt, drives microglia to produce a range of pro-inflammatory and neurotoxic mediators, such as TNF-α, IL-1α, IL-1β, IL-18, complement component C1q, ROS, Sema4D, EphrinB3, TGF-β, and VEGF-B, that enhance astrocytic inflammatory responses [17,18,19]. Similarly, elevated activation of NF-κB, iNOS, glutathione S-transferases and Akt in astrocytes leads to the release of signaling molecules, including TNF-α, IL-1β, IL-15, CCL2, C3, GM-CSF, NO, fibronectin, SFRP1, Wnt5a, C8γ, miR-137, IL-3, IL-33, and glutamate, which act on microglia to regulate their activation states under both physiological and pathological conditions [20,21,22,23,24,25,26].

Such complex bidirectional communication suggests that intracellular regulatory pathways play a critical role in the modulation of glial responses to injury and disease. Among these, the Hippo pathway and its downstream effectors YAP and TAZ (Yes-associated protein and transcriptional co-activator with PDZ-binding motif) have gained increasing attention for their role in regulating glial proliferation, polarization, and intercellular communication [27]. Given the multifaceted involvement of YAP/TAZ signaling in cellular mechanotransduction, glial activation, neuroinflammatory signaling, and tumorigenic transformation, its dysregulation may profoundly affect glial reactivity and the establishment of a microenvironment that supports tumor growth and immune evasion. Therefore, investigating the Hippo/YAP/TAZ axis in astrocyte–microglia interactions may provide novel insights into the molecular mechanisms that bridge neuroinflammation and oncogenesis in the CNS.

## 2. Structure and Core Components of the Hippo Pathway, Transcriptional Co-Activators and Hippo-Associated Signaling Pathways

### 2.1. Core Hippo Pathway Components and Regulation of YAP/TAZ Activity

**Figure 1:**
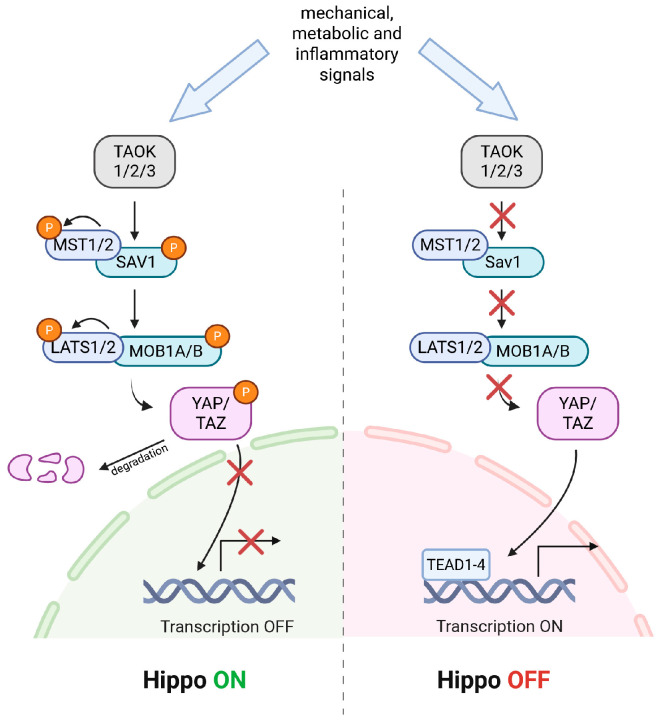
Structure and core components of the Hippo–YAP/TAZ signaling pathway. Mechanical, metabolic, and/or inflammatory signals regulate the Hippo core kinase cascade. In the Hippo ON state (**left**), TAOK1/2/3 activates MST1/2, which, via SAV1 and MOB1A/B, promotes activation of LATS1/2. Activated LATS1/2 phosphorylates YAP/TAZ, driving cytoplasmic sequestration and degradation and thereby suppressing TEAD1–4-dependent transcription (Transcription OFF). In the Hippo OFF state (**right**), reduced MST/LATS activity leaves YAP/TAZ unphosphorylated, allowing their nuclear translocation and binding to TEAD1–4 to induce gene programs controlling proliferation, migration, and survival (Transcription ON). Created in https://BioRender.com.

The Hippo signaling pathway was identified in genetic studies of organ development in *Drosophila melanogaster*, where loss of the Hippo kinase (Hpo) resulted in excessive tissue overgrowth [28]. This pathway is highly conserved in mammals. The core Hippo cascade consists of the serine/threonine kinases MST1/2 (Mammalian Sterile 20-like kinases 1/2, homologs of Hpo) and LATS1/2 (Large Tumor Suppressor 1/2, homologs of Warts) and two transcriptional co-activators, YAP and TAZ. MST1/2 kinases can be activated by phosphorylation at Thr183 and at Thr180, respectively, by serine/threonine protein kinases (TAOK1/2/3). MST1/2 can also undergo auto-activation through MST1/2 autophosphorylation by dimerization [29]. Their activity is further regulated by the adaptor protein SAV1 (Salvador family WW domain-containing protein 1), which facilitates the interaction between MST1/2 and LATS1/2. In turn, activation of LATS1/2 requires the adaptor proteins MOB1A/B (Mps One Binder kinase activator-like 1A/B), which form a complex with LATS1/2, enabling MST1/2-dependent phosphorylation of LATS1 at Thr1079 and LATS2 at Thr1041. MOB1A/B phosphorylation enhances its binding to LATS1/2 and initiates a cascade of activating autophosphorylation events within LATS kinases [30,31]. Additional regulatory input is provided by TAOK1/3, which can directly phosphorylate LATS1/2 at their hydrophobic motifs, and by MAP4K family kinases (MAP4K1/2/3/5 and MAP4K4/6/7), which act in parallel with MST1/2 to ensure robust LATS1/2 activation [32,33,34]. Activated LATS1/2 subsequently phosphorylate YAP and TAZ at conserved serine residues (Ser127 in YAP and Ser89 in TAZ), promoting their cytoplasmic sequestration via 14-3-3 proteins and ultimately their ubiquitin-mediated degradation [35]. When Hippo signaling is inactive, unphosphorylated YAP/TAZ translocate to the nucleus, where they interact primarily with TEAD1–4 transcription factors to drive the expression of genes controlling cell proliferation, migration, and survival (Figure 1) [36]. In this “Hippo OFF” state, YAP/TAZ function as potent growth regulators and are frequently hyperactivated in cancer [37]. Functionally, the Hippo–YAP/TAZ signaling integrates mechanical, metabolic, and inflammatory signals to maintain CNS homeostasis and coordinate glial responses to injury. Its dysregulation, through genetic alterations, epigenetic silencing, or aberrant mechanotransduction, contributes to pathological conditions such as fibrosis, neurodegeneration, and gliomagenesis [38]. Thus, understanding the structural and regulatory complexity of the Hippo–YAP/TAZ axis provides the molecular foundation for elucidating its roles in glial biology and tumor–immune interactions.

### 2.2. Crosstalk Between Hippo and Other Signaling Pathways

Importantly, the Hippo pathway interfaces extensively with other major signaling networks that regulate proliferation, apoptosis, differentiation, and immune responses. Among these, the Wnt/β-catenin, TGF-β/Smad, PI3K/Akt, and NF-κB pathways exhibit structured bidirectional interactions with Hippo–YAP/TAZ, forming an integrated regulatory signaling network that fine-tunes cellular behavior in response to environmental and mechanical stimuli.

#### 2.2.1. Crosstalk Between Hippo and Wnt/β-Catenin Signaling

Growing evidence indicates that Hippo signaling exerts strong regulatory control over the Wnt/β-catenin pathway. Hippo activation restricts β-catenin nuclear localization through phosphorylated YAP, thereby limiting Wnt transcriptional output [39]. Azzolin et al. further demonstrated that YAP/TAZ are integral components of the β-catenin destruction complex, associating with Axin, APC, GSK3β, and CK1 under Wnt-OFF conditions to promote β-TrCP-dependent β-catenin degradation while simultaneously preventing their own nuclear activity [40]. Upon Wnt activation, disassembly of the destruction complex leads to the concerted nuclear translocation of β-catenin and YAP/TAZ, enabling parallel activation of TCF/LEF (T-cell factor/lymphoid enhancer factor)- and TEAD-dependent gene expression programs. Wnt signaling also enhances YAP transcription via β-catenin/TCF complexes, forming a positive feedback loop [41]. YAP/TAZ additionally act as direct effectors of non-canonical Wnt pathways. Park et al. identified a β-catenin-independent Wnt–FZD/ROR–Gα12/13–Rho–LATS1/2 axis that activates YAP/TAZ and TEAD-mediated transcription. Notably, WNT5A/B and WNT3A were shown to stimulate YAP/TAZ nuclear accumulation independently of LRP5/6 and β-catenin, positioning YAP/TAZ as central integrators of alternative Wnt signaling outputs related to cytoskeletal dynamics, migration, and tissue remodeling [42]. Moreover, in astrocytes, YAP deletion reduces EAAT2 expression and impairs glutamate uptake, leading to neurotoxicity and cognitive impairment. Wnt3a-driven β-catenin activation enhances YAP signaling and restores EAAT2 expression, demonstrating cooperative YAP–Wnt activity in maintaining astrocyte-mediated neuroprotection [43]. Dysregulated Wnt/β-catenin signaling has further been implicated in neuroinflammation, blood–brain barrier impairment, and glial dysfunction in Alzheimer’s disease [44].

#### 2.2.2. Crosstalk Between Hippo and TGF-β/SMAD Signaling

Extensive crosstalk also occurs between Hippo and the TGF-β/SMAD axis, a central regulator of EMT, fibrosis, and cell fate determination. YAP has been shown to form transcriptional complexes with TEAD4 and SMAD3 on the CTGF promoter to potentiate TGF-β-driven extracellular matrix production [45]. Moreover, RASSF1A, a Hippo scaffold component, has been identified as a key regulator of YAP–SMAD interaction. Pefani et al. demonstrated that TGF-β stimulation induces RASSF1A recruitment to TGF-β receptor I, followed by ITCH-mediated degradation, enabling YAP–SMAD2 association and SMAD2 nuclear translocation, thereby amplifying TGF-β transcriptional output [46]. Importantly, this crosstalk is not restricted to cancer models but is also relevant in neuroinflammatory and glial contexts. In an experimental autoimmune encephalomyelitis model, astrocyte-specific YAP deletion resulted in marked suppression of TGF-β signaling, increased inflammatory infiltration, and exacerbated demyelination, while pharmacological activation of TGF-β partially mitigated these defects [47]. Collectively, these findings suggest a hierarchical organization in which SMAD signaling can direct YAP/TAZ transcriptional activity, with β-catenin exerting more context-dependent downstream effects [48].

#### 2.2.3. Crosstalk Between Hippo and PI3K/Akt Signaling

Interactions between Hippo and PI3K/Akt signaling further connect growth factor-dependent metabolic regulation with mechanotransduction. Early genetic studies in *Drosophila* showed that loss of Hippo pathway components leads to increased Akt levels and enhanced Akt phosphorylation, whereas Hippo activation suppresses Akt expression in a YAP-dependent manner, positioning Akt downstream of Hippo–YAP signaling in growth control [49]. Conversely, Akt signaling can modulate Hippo pathway output. Borreguero-Muñoz et al. demonstrated that nutritionally induced PI3K–PDK1–Akt activation promotes YAP nuclear accumulation in response to mechanical stimuli, establishing the Hippo as an integrator of insulin/IGF-1 and biomechanical signals [50]. YAP/TAZ can also enhance PI3K/Akt activity by promoting insulin receptor substrate 2 transcription, generating a feed-forward loop that contributes to metabolic dysregulation and tumorigenesis [51]. In mammalian systems, reciprocal feedback has also been described, whereby modulation of YAP activity alters PTEN and AKT phosphorylation status, further reinforcing bidirectional control between these two pathways [52].

#### 2.2.4. Crosstalk Between Hippo and NF-κB Signaling

The Hippo pathway also intersects with the NF-κB inflammatory cascade. Pro-inflammatory cytokines such as TNF-α activate TAK1, promoting YAP/TAZ degradation while stimulating NF-κB-dependent transcription [53]. Conversely, NF-κB/p65 can disrupt the YAP–LATS1 interaction, reducing YAP phosphorylation and facilitating its nuclear accumulation [54]. Hippo core kinases additionally regulate innate immune signaling upstream of NF-κB activation [55]. Depending on context, YAP may either enhance NF-κB-mediated cytokine production [56] or inhibit NF-κB transcriptional activity through competitive interactions with p65 binding partners [57]. Moreover, mechanical cues can activate an LATS1/2–PKCζ–NF-κB axis, linking Hippo signaling with mechanoinflammatory gene expression [58].

Together, these findings position the Hippo–YAP/TAZ pathway as a central signaling integrator that coordinates developmental, mechanical, metabolic, and immune cues through structured interactions with Wnt, TGF-β, PI3K/Akt, and NF-κB pathways. Rather than functioning as isolated modules, these pathways form a highly interconnected regulatory network in which YAP/TAZ act as context-dependent transcriptional hubs, fine-tuning cellular plasticity under physiological conditions while driving pathological reprogramming during neuroinflammation and tumorigenesis.

## 3. Hippo Signaling in Astrocytes and Microglia: From Inflammation to Neurodegeneration

### 3.1. Hippo Signaling in Microglial Inflammatory Polarization, Cytoprotection, and Mechanosensing

In microglia, MST1 activation promotes pro-inflammatory polarization and apoptotic signaling, thereby contributing to early neuroinflammatory cascades. Tian et al. [59] demonstrated that MST1 enhances microglial apoptosis by promoting dynamin-related protein 1-mediated mitochondrial fission and activating the JNK pathway. Conversely, inhibition of MST1/2 activity has been associated with a shift toward an anti-inflammatory phenotype. For instance, malibatol A, an inhibitor of MST1 phosphorylation, reduced the expression of pro-inflammatory cytokines TNF-α, IL-1β, and IL-6 in oxygen–glucose-deprived BV-2 microglia. This was accompanied by elevated levels of Ym1, CD206, IL-10, and TGF-β, markers of an alternative (M2-like), neuroprotective activation state [60]. Notably, MST1/2 activity does not uniformly promote inflammation across myeloid populations. In macrophages, MST1/2 signaling appears to support M2 polarization through STAT6 and MEK/ERK pathways, whereas its inhibition enhances YAP-driven M1-type inflammatory responses [61], highlighting a cell-type-specific regulatory role for Hippo signaling in innate immunity. These contrasting effects suggest that MST1/2 function is highly context-dependent, orchestrating a dynamic balance between reparative and defensive immune responses.

Accumulating evidence further indicates that YAP/TAZ activation supports neuroprotective and reparative functions in microglia. In LPS-stimulated BV-2 cells, TAZ overexpression reduced IL-1β, IL-6, and TNF-α secretion and lowered oxidative stress. These antioxidative effects were associated with increased activity of SOD, CAT, and GPX, elevated GSH levels, and an improved GSH/GSSG ratio, largely through Nrf2-dependent mechanisms. TAZ also inhibited mitochondrial permeability transition pore opening and cytochrome c release, repressing Bax while inducing Bcl-2, thereby exerting anti-apoptotic activity [62]. Identification of a TEAD-binding site in the Nrf2 promoter established a direct transcriptional link between TAZ and Nrf2.

Microglial survival under inflammatory conditions is also supported by Hippo-dependent metabolic adaptations. One study described a MAPK–ERK–YAP–BNIP3 axis that enhances mitophagy and protects against TNFα-induced cell death [63]. Expanding this metabolic dimension, recent work identified the Hippo-family kinase NDR2 as a regulator of glycolytic reprogramming and cytokine signaling, underscoring the immunometabolic functions of Hippo signaling in microglia [64]. In addition, recent evidence in AD further extends the pathogenic role of MST1 beyond apoptotic signaling to inflammatory cell death. In 5xFAD mice and Aβ-treated BV2 cells, MST1 activation was associated with abnormal microglial activation, pro-inflammatory cytokine production, and enhanced pyroptotic signaling, whereas microglia-specific MST1 knockdown suppressed the DPP8/NLRP1/Caspase-1/GSDMD-N axis and alleviated neuroinflammation, neurodegeneration, and cognitive deficits, identifying this pathway as a novel mechanism of chronic microglial inflammatory injury in AD [65].

In parallel, CNS injury induces the formation of fibrotic and glial scars that remodel the extracellular milieu, with stromal fibroblasts and scar-associated astrocytes depositing collagens, fibronectin and proteoglycans and thereby altering local tissue mechanics [66]. These ECM changes spatially and temporally coincide with the emergence of pro-inflammatory microglial subtypes and with downregulation of the Gas6/Axl axis in both microglia and astrocytes, creating a niche that favors inflammatory polarization. By suppressing astrocytic YAP activation, exogenous Gas6 reduces astrocyte-driven NF-κB and JAK–STAT signaling and thereby limits the propagation of pro-inflammatory programs to microglia, linking ECM-shaping processes to Hippo-dependent regulation of microglial reactivity [67]. Mechanical cues further regulate microglial phenotype through YAP/TAZ. Hu et al. [68] showed that Fascin-1, an F-actin-bundling protein, promotes microglial migration and functional recovery after spinal cord injury by upregulating TAZ expression and facilitating its nuclear translocation. Similarly, Dudiki et al. [69] reported that genetic depletion of the cytoskeletal adaptor kindlin-3 impairs membrane tension and disrupts YAP nuclear localization, highlighting the biomechanical sensitivity of this pathway. This was further validated in a stiffness-controlled culture system, where YAP translocated to the nucleus in response to ECM rigidity and activated TEAD-dependent transcription, increasing IL-10 while suppressing pro-inflammatory gene expression, thus fine-tuning microglial reactivity to biomechanical context [70].

### 3.2. Hippo Signaling in Astrocyte Reactivity, Neuroprotection, and Senescence

In astrocytes, Hippo signaling plays a multifaceted role in regulating reactive gliosis and neuroprotection. One mechanism involves inhibition of TANK-binding kinase 1 (TBK1) signaling, which suppresses astrocyte-mediated inflammation upon YAP inhibition [71]. Complementary findings suggest that YAP ubiquitination via PPM1B, promoted by the compound icariin, reduces astrocyte reactivity and supports functional recovery after spinal cord injury [72]. In addition, the miR-540-3p–SIX4–YAP1 axis has been identified as a critical regulator of astrocyte activation, where downregulation of this microRNA limits reactive gliosis and enhances neurological recovery [73]. However, the YAP function in astrocytes is highly context-dependent. Under chronic neuroinflammatory and aging conditions, prolonged TNF-α exposure suppresses YAP expression, leading to astrocyte senescence, reduced plasticity, and impaired neuroprotection [74]. Conversely, restoring YAP activity can be beneficial: its activation enhances expression of EAAT2, the main glutamate transporter, and promotes β-catenin-mediated transcription, reducing extracellular glutamate and protecting neurons from excitotoxicity [43,75,76]. YAP also restrains reactive astrogliosis by upregulating SOCS3, a negative regulator of the JAK–STAT3 pathway, thereby limiting astrocyte proliferation, hypertrophy, and glial scar formation following CNS injury [77].

ECM-dependent regulation adds another layer of context specificity to astrocytic Hippo–YAP signaling. After injury, astrocytes encounter a dynamically remodeled extracellular niche in which ECM composition and tissue mechanics change. These cues are transduced via cytoskeletal remodeling at astrocytic processes and end-feet that interface with the perivascular basement membrane and the neurovascular unit [78]. In a 3D injury-relevant hydrogel model with tunable stiffness, matrix softening initiated astrocytic activation with increased GFAP and IL-1β, whereas matrix stiffening reverted this phenotype, demonstrating that mechanical ECM cues can drive reversible state transitions [79]. Importantly, these stiffness-driven transitions were coupled to Hippo output. Astrocytes in stiffer matrices exhibited higher YAP expression and nuclear localization, while softening decreased YAP and coincided with a reactive phenotype. YAP knockdown further amplified GFAP induction, supporting YAP as a negative regulator of astrogliosis [79]. Mechanistically, ECM-derived mechanical inputs can be transmitted through adhesion and mechanotransduction pathways, including integrin-associated signaling and FAK, which converge on Rho GTPase-dependent actomyosin remodeling and thereby regulate YAP/TAZ nuclear translocation [79,80]. In vivo, astrocytic YAP is also modulated by injury-associated extracellular signals that shape the scar microenvironment. Gas6/Axl signaling suppressed YAP activation and nuclear translocation during cytotoxic astrocyte polarization, which attenuated astrocyte–microglia inflammatory crosstalk after spinal cord injury [67]. Finally, ECM-YAP coupling is likely reinforced by pathway crosstalk, since YAP and TAZ integrate with TGF-β/SMAD and Wnt/β-catenin signaling, which are major regulators of ECM remodeling and profibrotic programs [81]. In addition, astrocytic YAP has been discussed in neuroinflammatory demyelinating settings via TGF-β-linked mechanisms [82].

Importantly, YAP/TAZ also participates in the control of astrocyte cell cycle states. YAP deficiency in astrocytes reduces cell proliferation and promotes a premature senescence-like phenotype, which was accompanied by increased SA-β-Gal activity, upregulation of p16, p21, p53, and NF-κB-associated senescence markers, and reduced Lamin B1. Moreover, YAP knockout resulted in reduced expression of cyclin-dependent kinase 6 (CDK6), involved in the G1 phase progression and G1/S transition of the cell cycle [83]. Thus, in chronic neurodegenerative contexts, suppression of YAP may contribute not only to reactive dysfunction but also to loss of astrocytic renewal capacity and progressive entry into a senescent state.

### 3.3. Context-Dependent Roles of Hippo Signaling in Glial Responses

In summary, the consequences of YAP/TAZ activation or inhibition vary substantially depending on cell type, microenvironment, and disease stage. In microglia, YAP/TAZ activation is generally associated with anti-inflammatory, antioxidative, and cytoprotective responses, although the exact outcome remains injury-context-dependent. In astrocytes, however, the effects are more complex: YAP activity can support reactive remodeling, proliferation, and scar-associated responses in some acute injury settings, whereas its loss or sustained downregulation under chronic or degenerative stress impairs neuronal support, metabolic homeostasis, and tissue protection (Table 1). Importantly, these context-dependent effects are likely further shaped by disease-specific changes in extracellular matrix composition and stiffness, which influence YAP/TAZ activity through cytoskeletal tension and crosstalk with pathways such as TGF-β/SMAD and Wnt/β-catenin. These findings also suggest that YAP and TAZ may not be fully interchangeable. In the microglial studies discussed here, TAZ has been more directly linked to antioxidant and anti-inflammatory transcriptional programs, whereas YAP has more often been associated with mechanosensitive responses, mitophagy-related survival pathways, and nuclear signaling triggered by cytoskeletal tension or matrix rigidity. Although these distinctions remain incompletely resolved, they raise the possibility that YAP and TAZ differentially tune glial state transitions depending on the inflammatory and mechanical properties of the local CNS microenvironment. Taken together, these findings highlight that therapeutic targeting of Hippo signaling in the CNS will likely require a context-specific strategy that accounts for cell type, disease stage, and the integrated inflammatory, metabolic, and biomechanical cues determining whether glial responses become neuroprotective or neurotoxic.

## 4. Hippo Signaling in Glial Tumorigenesis

### 4.1. Hippo Signaling in Glioma Initiation, Stemness, and Tumor Cell Plasticity

The Hippo signaling pathway has emerged as a critical player in the pathogenesis of gliomas. In glioblastoma (GBM), the most aggressive glial tumor, multiple molecular alterations disrupt the Hippo cascade, promoting malignant transformation, growth, and therapy resistance. A foundational insight into this pathway came from the identification of TAZ as a master regulator of the mesenchymal (MES) transcriptional subtype of GBM. TAZ is markedly upregulated in MES glioma stem cells, where it induces invasive behavior, self-renewal, and lineage-specific differentiation through TEAD2-dependent transcription. Importantly, TAZ mutants lacking TEAD-binding ability fail to induce MES identity, underscoring the essential role of this interaction [84].

At the upstream regulatory level, epigenetic silencing of Hippo core components represents a recurrent mechanism in diffuse gliomas. Promoter hypermethylation of RASSF1A, LATS2, and MST1 has been reported in glioma samples, indicating that repression of Hippo pathway members may release constraints on tumor growth and progression. In particular, co-silencing of RASSF1 and LATS2 identifies a clinically distinct subgroup of IDH-mutant tumors with a favorable prognosis [85]. Additional evidence comes from TGF-β-mediated induction of DNMT1, which suppresses MST1 expression and enhances glioma proliferation and invasiveness, whereas pharmacological inhibition of DNMT1 restores MST1 expression and reverses these oncogenic effects [86]. MST1 also exerts tumor-suppressive activity through the FOXO3a–SIRT6 axis. MST1 overexpression increases SIRT6 levels, reduces glioma cell viability, and promotes apoptosis, whereas SIRT6 knockdown markedly attenuates these effects, establishing the MST1–FOXO3a–SIRT6 pathway as a suppressive module in glioma cells [87].

Overall, these findings indicate that Hippo pathway dysregulation in glioma is not limited to loss of growth restraint but also supports stem-like states, mesenchymal transition, and tumor cell plasticity.

### 4.2. Hippo Signaling in the Glioma Microenvironment and Therapy Resistance

Beyond tumor-intrinsic growth control, Hippo effectors also shape the glioma immune microenvironment. YAP contributes to the creation of an immunosuppressive tumor microenvironment by regulating PD-L1 expression and promoting recruitment of regulatory T cells and tumor-associated macrophages. Pharmacological inhibition of YAP nuclear translocation using moxidectin disrupts these immune-evasive mechanisms and suppresses intracranial tumor growth [88]. YAP also regulates tumor immunometabolism by enhancing glycolytic and glutaminolytic programs, thereby supporting both energy production and immune escape [89].

Therapy resistance represents another major functional dimension of Hippo dysregulation in GBM. In temozolomide-resistant tumors, P4HA1-mediated prolyl hydroxylation stabilizes YAP by preventing its ubiquitin-dependent degradation, leading to increased expression of ECM components such as COL1A1. This ECM remodeling reinforces chemoresistance and cellular survival, and disruption of the P4HA1–YAP axis sensitizes GBM cells to treatment [90]. Hippo signaling is also modulated by ubiquitin ligases such as NEDD4L. DNMT1-dependent methylation of the NEDD4L promoter reduces its expression, thereby promoting YAP stabilization and VEGFA-driven angiogenesis. The lncRNA BDNF-AS counteracts this process by blocking DNMT1 recruitment to the NEDD4L promoter and preserving NEDD4L-mediated tumor suppression [91].

The oncogenic activity of YAP/TAZ is tightly linked to TEAD transcription factors. TEAD3, in particular, has been identified as a critical cofactor in glioma stem-like cells and correlates with adverse clinical outcomes. Although TEAD3 inhibition does not directly impair proliferation, it disrupts sterol metabolism, a metabolic dependency required for tumor maintenance, thereby revealing a functionally relevant TEAD-linked vulnerability [92].

Genomic rearrangements involving YAP1 represent an additional mechanism of pathway dysregulation in glial tumors. YAP1 fusions, previously described in ependymomas and other CNS tumors, drive constitutive activation of downstream transcriptional programs and may bypass upstream Hippo control mediated by MST1/2 and LATS1/2. These rearrangements may therefore define biologically distinct tumor subsets with unique clinical trajectories [93,94].

### 4.3. Mechanical Regulation of Hippo Signaling in GBM

ECM remodeling in GBM likely provides an additional layer of Hippo pathway activation through mechanotransduction. GBM-associated accumulation and reorganization of collagens, fibronectin, hyaluronan, and tenascin-C increase local tissue stiffness and activate integrin- and focal-adhesion-dependent signaling pathways [95,96]. Based on general mechanotransduction principles, such biomechanical inputs are likely to promote nuclear YAP/TAZ activity [97]. In turn, this may reinforce mesenchymal transition, invasive behavior, and therapy resistance, consistent with studies linking matrix-dependent biomechanics to GBM infiltration and YAP/TAZ-driven cell-state plasticity [98,99].

Upstream signaling pathways further converge on YAP/TAZ activation. Rho GTPase-driven cytoskeletal remodeling promotes YAP activation and enhances glioma cell motility and stemness [100]. Similarly, the PI3K–AKT–mTOR axis interacts with Hippo components, linking metabolic adaptation and growth factor cues to glioma aggressiveness [101]. YAP/TAZ also cooperate with MYC-driven oncogenic programs: TEAD–YAP complexes regulate MYC target genes, supporting proliferation and survival in glioma stem-like cells. Pharmacological disruption of YAP–TEAD interactions, therefore, impairs tumorigenic capacity and reveals a tractable vulnerability within the Hippo–MYC signaling network [102].

In summary, in the tumor context, Hippo signaling is coupled much more directly to proliferative competence, stemness, and cell-state plasticity than in non-neoplastic glia. Thus, one major distinction between neurodegenerative and neoplastic settings is that the same Hippo effectors that regulate stress adaptation and homeostatic responses in astrocytes and microglia can, in glioma, become integrated into sustained proliferative and stemness-associated programs. This also illustrates that YAP and TAZ are not necessarily functionally redundant in glioma biology. Whereas TAZ appears particularly prominent in enforcing mesenchymal glioma stem-cell identity [84], YAP has been more broadly implicated in immune evasion, metabolic adaptation, angiogenesis, ECM-linked mechanotransduction, and therapy resistance [90,99]. Together, these findings identify Hippo signaling not only as a central regulatory axis in glial tumorigenesis but also as a source of therapeutic vulnerabilities across multiple dimensions of GBM biology.

## 5. Therapeutic Perspectives and Research Directions

### 5.1. Selective Targeting of Hippo–YAP/TAZ Signaling in Glioblastoma

**Figure 2:**
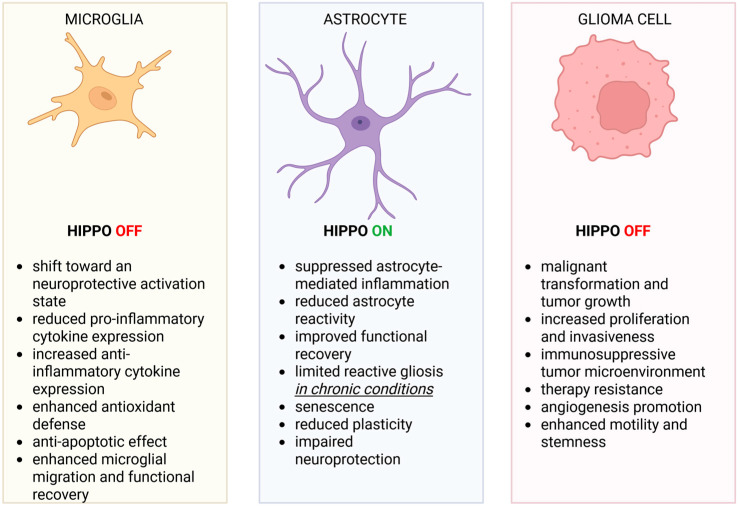
Cell-type-specific consequences of Hippo pathway status. This schematic summarizes the distinct functional outcomes associated with Hippo signaling states across microglia (**left**), astrocytes (**middle**) and glioma cells (**right**). Created in https://BioRender.com.

The context-dependent roles of Hippo signaling in the CNS indicate that uniform modulation of YAP/TAZ, whether through global activation or inhibition, is unlikely to be consistently safe or therapeutically effective. Transcriptional programs that support tissue repair and limit inflammation in glial cells may, in other cellular contexts or disease stages, promote glioma stem-like phenotypes, infiltrative growth, or immune evasion (Figure 2). Consequently, therapeutic strategies will likely require context-gated targeting defined by cell type (tumor cells vs. microglia or astrocytes), disease phase (acute injury, chronic neuroinflammation, or established tumor), spatial niche (hypoxic, perivascular, or invasive-edge regions), and molecular subtype. Single-cell profiling supports this view by demonstrating that YAP/TAZ regulate glioblastoma plasticity and that inhibition may be most effective in tumor compartments capable of state transitions rather than uniformly across all cells [99]. Emerging therapeutic strategies are now seeking to exploit these context-specific dependencies. Verteporfin has been repurposed as a functional YAP–TEAD inhibitor in preclinical GBM models, leveraging an established regulatory and pharmacological profile [103,104]. In parallel, next-generation TEAD inhibitors, targeting the central pocket or palmitoylation-dependent activation, have entered clinical development in non-CNS cancers, offering candidates for translation into neuro-oncology pending confirmation of CNS penetration, tolerability, and pharmacodynamic effect [105,106,107,108]. Another strategy is to mimic endogenous YAP antagonists. VGLL4-derived Tondu-domain peptides compete with YAP for TEAD binding and repress YAP-dependent transcription [109]. Although delivery of peptides to brain tumors remains challenging, these findings offer a structural framework for the development of stapled peptides, macrocycles, or small-molecule PPI inhibitors with CNS-penetrant properties. Recent data further support YAP1 as a therapeutic target in GBM. Both YAP1 siRNA and the YAP1 inhibitor CA3 (CIL56) reduced viability, migration, invasion, and spheroid formation in glioblastoma cells, consistent with suppression of tumorigenic and stem-like properties. These effects were accompanied by G0/G1 arrest in T98G cells, reversal of EMT-related features, and broad suppression of YAP/TEAD-, β-catenin-, MAPK-, and mTOR-related programs, while cancer stem cells also showed increased ferroptosis-related susceptibility [110].

### 5.2. Combination Strategies, Immune Modulation, and Translational Delivery Challenges

However, targeting the Hippo axis as monotherapy is unlikely to provide a lasting therapeutic effect. Studies in non-CNS demonstrate that TEAD palmitoylation inhibitors can trigger adaptive resistance through pathway rewiring, supporting the rationale for combination approaches. Mechanistic analyses have identified actionable co-dependencies, most notably MAPK pathway inhibition, that enhance antitumor efficacy when paired with TEAD blockade [111]. This aligns with GBM findings showing that MEK–ERK signaling modulates YAP/TAZ transcriptional output and that MEK–ERK inhibitors reduce nuclear YAP/TAZ abundance while altering the immune compartment [88]. Identifying therapeutic combinations that prevent compensatory transcriptional activation and sustain suppression of invasive, recurrence-associated cell states is therefore a central objective.

Hippo effectors also regulate multiple immune-evasion pathways, including PD-L1 expression and macrophage-recruiting programs. Direct transcriptional regulation of PD-L1 by YAP/TEAD complexes has been demonstrated in a non-glioma context [112,113], providing a rationale to test whether YAP/TEAD inhibition can increase the responsiveness of immunologically “cold” glioblastomas to immune checkpoint blockade, particularly when combined with treatments that promote tumor-antigen release or activate innate immunity. In glioma-relevant studies, YAP activation increases expression of macrophage-attracting chemokines such as CSF1 and CCL2, linking tumor-intrinsic Hippo signaling with the abundance and polarization of tumor-associated macrophages [114]. Rational combination strategies may therefore be tailored based on immune texture: in macrophage-enriched tumors, pairing Hippo-pathway inhibitors with macrophage-targeting therapies; in lymphocyte-excluded tumors, pairing them with approaches that enhance antigen presentation and T-cell priming [88].

A major translational constraint is that YAP/TAZ can be protective in certain inflammatory contexts in microglia and astrocytes. Therapeutic development must therefore prioritize tumor-selective exposure and state-selective targeting, exploiting tumor-specific dependencies on TEAD-driven transcription while minimizing disruption of physiological Hippo signaling in non-malignant glia. Potential solutions include tumor-targeted nanoparticles, convection-enhanced delivery to invasive margins, and prodrug designs activated by tumor-associated enzymes or hypoxia. Early incorporation of BBB-relevant models, including BBB-on-a-chip platforms, may improve translational success by enabling permeability and toxicity assessment under controlled barrier conditions [115].

### 5.3. Hippo Signaling as a Therapeutic Target in Neuroinflammation and Neurodegeneration

Beyond oncologic applications, modulation of Hippo signaling in glial cells has significant therapeutic potential in conditions where chronic inflammation drives neurodegeneration. In astrocytes, YAP downregulation, observed in aging brains and AD models, promotes senescence, release of pro-inflammatory cytokines (IL-6 and IL-8), and cognitive decline, whereas restoration of YAP activity or MST1/2 inhibition reverses these effects and improves behavioral outcomes [83]. Hyperactivation of MST1/2 similarly promotes oxidative stress and mitochondrial damage and accelerates neuronal degeneration and cognitive deficits [116], identifying these kinases as tractable therapeutic nodes. Moreover, neuronal studies deepen this rationale. Tanaka et al. identified early YAP loss as a key determinant of YAP-dependent necrosis in pre-symptomatic AD, caused by YAP sequestration into cytoplasmic Aβ aggregates. AAV-mediated delivery of the neuroprotective isoform YAP-deltaC, capable of antagonizing YAP–p73 pro-death signaling, suppressed early neuronal necrosis, reduced downstream Aβ burden, and improved cognitive outcomes in AD models [117]. These findings support Hippo-pathway modulation, through MST1/2 inhibition, restoration of YAP activity in astrocytes, or neuronal reinforcement of YAP-dependent survival signaling, as a promising strategy to interrupt the transition from chronic glial inflammation to neurodegeneration. Importantly, these therapeutic implications differ fundamentally from those in glioma. In neurodegenerative contexts, the desired outcome is not sustained cell-cycle activation, but restoration of protective glial programs, limitation of senescence, and preservation of metabolic and trophic support. This distinction reinforces the need to interpret YAP/TAZ signaling in a cell state-specific manner, as proliferative outputs that are advantageous for tumor growth may be absent, attenuated, or even maladaptive in chronically stressed astrocytes and microglia. Current therapeutic strategies targeting the YAP/TAZ pathway are summarized in Table 2.

## 6. Conclusions

Hippo–YAP/TAZ signaling has emerged as a central but highly context-dependent regulatory axis at the intersection of glial inflammation, neurodegeneration, and glioma biology. Across astrocytes and microglia, this pathway integrates inflammatory, metabolic, and mechanical cues to shape cell state transitions that may either preserve CNS homeostasis or promote pathological reprogramming. In gliomas, dysregulated Hippo signaling supports stemness, immune evasion, and therapy resistance, whereas in chronic neuroinflammatory and neurodegenerative settings, loss of protective YAP/TAZ activity contributes to glial senescence, neuronal dysfunction, and disease progression. These dual and context-specific roles indicate that successful therapeutic targeting of the Hippo pathway will require carefully tailored, combination-based, and cell state-specific strategies that preserve beneficial functions in non-malignant glia while exploiting tumor-selective dependencies.

## Figures and Tables

**Table 1:** Effects of Hippo pathway modulation in astrocytes and microglia under physiological and pathological conditions.

Cell Type	Condition	Hippo Component Modulation	Main Effect	Functional Consequence	Ref.
Microglia	Inflammation	↓ MST1	Mst1 silencing suppresses JNK activation and Drp1 expression; reduces mitochondrial fragmentation; restores mitochondrial length; increases ATP production; lowers ROS; and decreases cytochrome c release, mPTP opening, caspase-3/9 activity and LDH release	Anti-inflammatory, anti-oxidative, anti-fission, and anti-apoptotic effects that preserve mitochondrial homeostasis and promote cell survival	[59]
		↑ TAZ	TAZ activation suppresses IL-1β, IL-6, and TNF-α; reduces ROS, MDA, and 8-OHdG; enhances Nrf2 transcription and nuclear translocation; increases anti-oxidative defenses (SOD, CAT, GPX, GR, GSH, and GSH/GSSG ratio); preserves ATP; inhibits mPTP opening and cytochrome c release; decreases caspase-3 and Bax; increases Bcl2; and represses NF-κB activation	Anti-inflammatory, anti-oxidative, mitochondrial-protective, and anti-apoptotic effects that attenuate microglia-mediated inflammatory injury	[62]
		↑ YAP	YAP activation upregulates Bnip3-related mitophagy; preserves mitochondrial membrane potential; reduces mPTP opening, HtrA2/Omi release, ROS overproduction, and caspase-9/caspase-3 activation; and improves ATP production, mitochondrial respiratory complex expression, and anti-oxidative defenses (GSH, SOD, and GPx)	Anti-apoptotic, mitochondrial-protective, anti-oxidative, and cytoprotective effects that protect cells from inflammatory injury	[63]
		↓ MST1	MST1 knockdown reduces DPP8/NLRP1/Caspase-1/GSDMD-N mediated microglial pyroptosis and inflammatory injury in AD	Anti-pyroptotic, anti-inflammatory, and neuroprotective effects that attenuate AD progression	[65]
	Oxygen–glucose deprivation	↓ MST1/2	MST1/2 inhibition decreases apoptosis; increases cell viability; downregulates M1-associated markers: MCP-1, IL-1, and TNF-α; and upregulates M2-associated markers (Ym-1, CD206, IL-10, and TGF-β)	Anti-apoptotic, anti-inflammatory, and pro-reparative effects that promote M2 microglial polarization and protect cells from OGD injury	[60]
	Spinal cord injury	↑ TAZ	TAZ activation promotes microglial migration, enhances aggregation of microglia around the lesion core, and increases microglial scar formation	Pro-migratory, scar-forming, and neuroprotective effects that support microglial scar formation and promote neurological recovery after SCI	[68]
	Mechanical cues	↓ YAP	Loss of Kindlin3 reduces membrane-to-cortex attachment, lowers membrane tension and cell stiffness, increases membrane blebbing/ruffling, mislocalizes ERM proteins, and decreases YAP nuclear localization	Impaired mechanotransduction and defective membrane mechanics, leading to reduced YAP-dependent nuclear signaling	[69]
		↑ YAP	Increased matrix stiffness enhances YAP expression and nuclear localization, which is associated with elevated IL-10 secretion	Enhanced anti-inflammatory/immunoregulatory signaling and pro-proliferative effects of microglia on gliomas	[70]
Astrocytes	Spinal cord injury	↓ YAP	TBK1 inhibition suppresses YAP expression and nuclear translocation, reduces astrocyte proliferation and astrocyte-derived IL-1β and IL-6, decreases GFAP+ astrogliosis, and improves neuronal survival and locomotor recovery after SCI	Reduced neuroinflammation and improved functional recovery	[71]
		↓ YAP	Inhibition of YAP expression reduces Smad2/3 phosphorylation; inhibits GFAP/vimentin/Ki67-positive reactive astrocyte proliferation; increases PPM1B ubiquitination and nuclear translocation; and improves neuronal preservation, myelin integrity, gait and electrophysiological recovery after SCI	Reduced glial scarring and improved tissue and motor recovery	[72]
		↓ YAP	YAP inhibition inactivates reactive astrocytes, suppresses inflammatory cytokine secretion, preserves neurite length, decreases neuronal apoptosis, reduces glial scar formation, and improves locomotor recovery through the SIX4/YAP1 signaling axis	Anti-inflammatory, reactive astrocyte-suppressing, neuroprotective, and functional recovery-promoting effects in SCI	[73]
		↓ YAP	YAP inhibition is associated with premature astrocyte senescence, increased inflammation, ROS accumulation, and mitochondrial damage	Pro-senescent, pro-inflammatory, and dysfunction-promoting effects that worsen SCI outcome in aging	[74]
	Ischemic stroke	↑ YAP	YAP activation upregulates EAAT2 through β-catenin signaling, limits glutamate excitotoxicity, promotes astrocyte proliferation and glial scar formation, promotes astrocyte stemness, reduces neuronal apoptosis and inflammatory infiltration, and improves functional recovery	Neuroprotective and functional recovery-promoting effects in ischemic stroke	[75]
	Amyotrophic Lateral Sclerosis	↑ YAP	YAP activation upregulates β-catenin- and EAAT2-dependent glutamate clearance in astrocytes, limits glutamate excitotoxicity, preserves neurons, reduces TDP-43 pathology and inflammatory infiltration, supports astrocyte proliferation, and improves motor function	Neuroprotective and motor function-preserving effects in ALS	[76]
	Inflammation	↑ YAP	YAP activation induces SOCS3 (and SOCS1), restrains JAK/STAT inflammatory signaling, suppresses STAT3 hyperactivation, and prevents reactive astrocyte activation	Anti-inflammatory, reactive astrocyte-suppressing, and blood–brain barrier-protective effect	[77]
	Mechanical cues	↓ YAP	Soft matrices decrease YAP expression and nuclear localization, increase GFAP and IL-1β expression, promote astrocyte spreading and hypertrophic/reactive morphology, and induce astrogliosis	Reactive astrocyte-promoting, pro-inflammatory, and glial scar-promoting effects under low matrix stiffness	[79]
	Experimental autoimmune encephalomyelitis (EAE)/MS-related optic neuritis	↑ YAP	YAP activation promotes the proliferation of astrocytes and upregulates TGF-β signaling to prevent neuroinflammation in the optic nerve and retina of EAE mice, which further inhibits the demyelination of the optic nerve and the loss of RGCs in the retina	Anti-inflammatory, anti-demyelinating, retina-protective, and neuroprotective effects	[47]

↑, upregulation/increased activity; ↓, downregulation/decreased activity.

**Table 2:** Current and emerging therapeutic strategies targeting Hippo–YAP/TAZ signaling in CNS disease.

Therapeutic Strategy	Molecular Target/Mechanism	Representative Agent(s)	Disease Context	Refs.
Functional YAP–TEAD inhibition	Disruption of YAP–TEAD transcriptional activity	Verteporfin	GBM	[103,104]
Direct TEAD inhibition	Central pocket targeting/palmitoylation-dependent TEAD inhibition	Next-generation TEAD inhibitors	Non-CNS cancers; proposed for GBM	[105,106,107,108]
Mimicking endogenous YAP antagonists	Competitive inhibition of YAP–TEAD binding	VGLL4-derived Tondu-domain peptides	Non-CNS cancers; proposed for GBM	[109]
Direct YAP1 inhibition	Genetic or pharmacologic inhibition of YAP1	YAP1 siRNA; CA3 (CIL56)	GBM	[110]
Combination with MAPK pathway inhibition	Blocking adaptive resistance and compensatory transcriptional rewiring	TEAD inhibitor + MAPK/MEK inhibition	GBM and non-CNS cancers	[111]
Combination with immune checkpoint blockade	Counteracting YAP/TEAD-driven PD-L1 expression and immune evasion	YAP/TEAD inhibitor + anti-PD-1/PD-L1 strategies	Immunologically “cold” GBM	[112,113]
Combination with macrophage-targeting therapies	Targeting YAP-driven macrophage recruitment programs	Hippo-pathway inhibitors + TAM/macrophage-directed agents	Macrophage-enriched GBM	[114]
Tumor-selective delivery approaches	Improved selectivity and CNS exposure	Tumor-targeted nanoparticles, convection-enhanced delivery, hypoxia/enzyme-activated prodrugs	GBM	[115]
Upstream Hippo modulation	Inhibition of MST1/2 to restore protective glial states	Experimental MST1/2 inhibitors	Neuroinflammation/neurodegeneration	[83,116]
Neuronal reinforcement of YAP-dependent survival signaling	Counteracting YAP loss–associated neuronal death	AAV-mediated YAP-deltaC delivery	AD	[117]

## Data Availability

No new data were created or analyzed in this study. Data sharing is not applicable to this article.

## Abbreviations

The following abbreviations are used in this manuscript:

8-OHdG	8-hydroxy-2′-deoxyguanosine
AAV	adeno-associated virus
AD	Alzheimer’s disease
Akt	protein kinase B
ALS	amyotrophic lateral sclerosis
APC	adenomatous polyposis coli
BBB	blood–brain barrier
BDNF-AS	brain-derived neurotrophic factor antisense RNA
BNIP3	BCL2/adenovirus E1B 19 kDa-interacting protein 3
BV-2	microglial cell line
C1q	complement component C1q
C3	complement component C3
C8γ	complement component 8 gamma chain
CAT	catalase
CCL2	C-C motif chemokine ligand 2
CDK6	cyclin-dependent kinase 6
CK1	casein kinase 1
CNS	central nervous system
COL1A1	collagen type I alpha 1 chain
CTGF	connective tissue growth factor
DNMT1	DNA methyltransferase 1
Drp1	dynamin-related protein 1
EAAT1/2	excitatory amino acid transporter 1/2
EAE	experimental autoimmune encephalomyelitis
ECM	extracellular matrix
EGFR	epidermal growth factor receptor
EMT	epithelial–mesenchymal transition
ERK	extracellular signal-regulated kinase
FAK	focal adhesion kinase
FZD	Frizzled receptor
GBM	glioblastoma multiforme
GM-CSF	granulocyte–macrophage colony-stimulating factor
GPX	glutathione peroxidase
GSH	reduced glutathione
GSH/GSSG	reduced glutathione/oxidized glutathione ratio
GSK3β	glycogen synthase kinase 3 beta
HIF1α	hypoxia-inducible factor 1 alpha
Hpo	Hippo kinase
iNOS	inducible nitric oxide synthase
IDH	isocitrate dehydrogenase
IGF-1	insulin-like growth factor 1
IL-1α/1β/3/6/8/10/15/18/33	interleukin-1 alpha, 1 beta, -3, -6, -8, -10, -15, -18, -33
IRS2	insulin receptor substrate 2
ITCH	itchy E3 ubiquitin protein ligase
JAK–STAT3	Janus kinase–signal transducer and activator of transcription 3
JNK	c-Jun N-terminal kinase
LDH	lactate dehydrogenase
LPS	lipopolysaccharide
LATS1/2	large tumor suppressor 1/2
LRP5/6	low-density lipoprotein receptor-related protein 5/6
MAP4K	mitogen-activated protein kinase kinase kinase kinase
MAPK	mitogen-activated protein kinase
MDA	malondialdehyde
MEK	mitogen-activated protein kinase kinase
MES	mesenchymal
miR	microRNA
MOB1A/B	Mps one binder kinase activator-like 1A/B
mPTP	mitochondrial permeability transition pore
MST1/2	mammalian sterile 20-like kinases 1/2
MYC	MYC proto-oncogene
NDR2	nuclear Dbf2-related kinase 2
NEDD4L	neural precursor cell expressed developmentally downregulated 4-like
NF-κB	nuclear factor kappa B
NO	nitric oxide
Nrf2	nuclear factor erythroid 2-related factor 2
OGD	oxygen–glucose deprivation
P4HA1	prolyl 4-hydroxylase subunit alpha 1
PD-1	programmed cell death protein 1
PD-L1	programmed death-ligand 1
PDK1	phosphoinositide-dependent protein kinase 1
PI3K	phosphoinositide 3-kinase
PPI	protein–protein interaction
PKCζ	protein kinase C zeta
PPM1B	protein phosphatase, Mg^2+^/Mn^2+^-dependent 1B
PTEN	phosphatase and tensin homolog
RASSF1A	Ras association domain family member 1 isoform A
ROS	reactive oxygen species
ROR	receptor tyrosine kinase-like orphan receptor
SAV1	Salvador family WW domain-containing protein 1
SCI	spinal cord injury
Sema4D	semaphorin 4D
SFRP1	secreted frizzled-related protein 1
SIRT6	sirtuin 6
SIX4	sine oculis homeobox homolog 4
SMAD2/3	SMAD family member 2/3
SOCS3	suppressor of cytokine signaling 3
SOD	superoxide dismutase
TAOK1/2/3	thousand-and-one amino acid kinases 1/2/3
TAM	tumor-associated macrophage
TAZ	transcriptional co-activator with PDZ-binding motif
TBK1	TANK-binding kinase 1
TCF/LEF	T-cell factor/lymphoid enhancer factor
TEAD1–4	TEA domain transcription factors 1–4
TGF-α/β	transforming growth factor alpha/beta
TGF-βRI	transforming growth factor beta receptor I
TNF-α	tumor necrosis factor alpha
Treg	regulatory T cell
VGLL4	vestigial-like family member 4
VEGF-B	vascular endothelial growth factor B
VEGFA	vascular endothelial growth factor A
WNT3A	Wnt family member 3A
WNT5A/B	Wnt family members 5A and 5B
Wnt	Wingless/Integrated
YAP	Yes-associated protein
YAP-deltaC	C-terminally truncated YAP isoform
Ym1	chitinase-like protein 3
